# Monitoring of microbial dynamics in a drinking water distribution system using the culture-free, user-friendly, MYcrobiota platform

**DOI:** 10.1038/s41598-018-32987-x

**Published:** 2018-10-03

**Authors:** Stefan A. Boers, Emmanuelle I. Prest, Maja Taučer-Kapteijn, Aleksandra Knezev, Peter G. Schaap, John P. Hays, Ruud Jansen

**Affiliations:** 1 Department of Medical Microbiology and Infectious Diseases, Erasmus University Medical Centre Rotterdam (Erasmus MC), Rotterdam, The Netherlands; 2 PWNT, Andijk, The Netherlands; 3 Het Waterlaboratorium, Haarlem, The Netherlands; 4 Water Supply Company Noord-Holland PWN, Velserbroek, The Netherlands; 5 Department of Molecular Biology, Regional Laboratory of Public Health Kennemerland, Haarlem, The Netherlands

## Abstract

Drinking water utilities currently rely on a range of microbiological detection techniques to evaluate the quality of their drinking water (DW). However, microbiota profiling using culture-free 16S rRNA gene next-generation sequencing (NGS) provides an opportunity for improved monitoring of the microbial ecology and quality of DW. Here, we evaluated the utility of a previously validated microbiota profiling platform (MYcrobiota) to investigate the microbial dynamics of a full-scale, non-chlorinated DW distribution system (DWDS). In contrast to conventional methods, we observed spatial and temporal bacterial genus changes (expressed as operational taxonomic units - OTUs) within the DWDS. Further, a small subset of bacterial OTUs dominated with abundances that shifted across the length of the DWDS, and were particularly affected by a post-disinfection step. We also found seasonal variation in OTUs within the DWDS and that many OTUs could not be identified, even though MYcrobiota is specifically designed to reduce potential PCR sequencing artefacts. This suggests that our current knowledge about the microbial ecology of DW communities is limited. Our findings demonstrate that the user-friendly MYcrobiota platform facilitates culture-free, standardized microbial dynamics monitoring and has the capacity to facilitate the introduction of microbiota profiling into the management of drinking water quality.

## Introduction

Drinking water distribution systems (DWDSs) are complex ecosystems where microorganisms can actively grow and reproduce. In fact, several studies have shown that the bacterial composition within drinking water (DW) samples are highly diverse^1,2^, with total cell concentrations that typically range from 1,000 to 100,000 bacterial cells per milliliter^3^. Importantly, the presence of (opportunistic) pathogenic bacteria within DW may present an emerging public-health risk^4^. Moreover, certain bacteria may also cause operational problems within DWDSs due to bacterial induced corrosion of iron pipes^5^, or produce metabolites that affect the taste, odor and color of DW^6^. To reduce the risks of bacterial growth, DW utilities employ a combination of several treatment processes to minimize the number of microorganisms, as well as the microbial growth supporting nutrients, in the DW produced. These treatments commonly involve primary disinfection processes e.g. chlorination, ozonation, or UV/H_2_O_2_ advanced oxidation, which are also used for oxidation of natural organic matter, combined with filtration processes, such as active carbon, rapid or slow sand filtration, soil infiltration, or membrane filtration methods^7^. However, despite these efforts, eliminating all microorganisms and nutrients during treatment is impossible using current treatment processes, meaning that bacterial growth in the DWDS can occur and that DW utilities are compelled to monitor microbiological changes during DW treatment and distribution.

Current microbiological characterization of DW relies heavily on conventional culturing techniques such as heterotrophic plate counts (HPC) and selective plating for *Aeromonas* spp., *Legionella* spp. and fecal indicator bacteria. Importantly however, these culture-based methods are time-consuming and it is well-known that they often detect only a small proportion of the total microbial population present in DWDSs^8^. Therefore, multiple culture-independent methods have been developed over the past decade to overcome these limitations. Most notably, flow cytometry (FCM) has emerged as a promising tool for the rapid assessment of DW quality that enables the detection and quantification of relevant microbial dynamics throughout a DWDS with high sensitivity^9^. Although FCM is useful for counting the total and viable number of bacterial cells throughout a DWDS, it does not generate taxonomic information about the microbial composition within DWDSs. The identification and quantification of bacterial taxa is required in order to adequately evaluate the complex nature of microbial communities within DW samples and to determine whether potentially (opportunistic) pathogenic bacteria are present within the DWDS. In contrast to FCM, microbiota profiling methods using 16S rRNA gene next-generation sequencing (NGS) techniques are able to differentiate the composition of microbial communities on a taxonomic level and these methods have already been applied to DWDSs^2,10,11^. Importantly however, obtaining accurate 16S rRNA gene profiles requires careful consideration of (often overlooked) PCR amplification biases and bacterial DNA contamination that can be introduced during the many steps of sample processing and sequencing^12^. These inevitable biases frustrate the accurate validation of current 16S rRNA gene NGS methods and, consequently, impedes the transition of these powerful tools from research into industrial diagnostic practice.

Recently, the authors published a validated micelle PCR/NGS (micPCR/NGS) methodology that significantly reduces PCR amplification biases in microbiota profiles via the clonal amplification of targeted 16S rRNA gene molecules^13^. The micPCR/NGS method drastically reduces chimera formation compared with traditional 16S rRNA gene NGS methods and prevents PCR competition due to unequal amplification rates of different 16S rRNA gene template molecules. This is of particular importance for the accurate analysis of microbial compositions within high-diversity samples, such as DW samples, as these samples are more vulnerable to chimera formation and PCR competition compared to low-diversity samples^13^. Further, by adding an internal calibrator (IC) to the micPCR/NGS methodology, we are able to quantify the absolute abundances of the bacterial operational taxonomic units (OTUs) detected within the samples under investigation, which enables the subtraction of any non-sample associated contaminating bacterial DNA that is invariably present in the laboratory environment and chemicals/reagents mixes used via the processing of negative extraction control (NEC) samples^14^. Therefore, the micPCR/NGS methodology possess a much higher accuracy and a lower limit of detection (LOD) compared with traditional 16S rRNA gene NGS methods that allows for the accurate detection of minor microbial variations within DWDSs. Another problem associated with the introduction of 16S rRNA gene NGS methodologies into industrial processes involves the complexity associated with establishing a 16S rRNA gene analysis workflow that can be operated by non-bioinformatics educated users. To overcome this limitation, we previously developed a dedicated bioinformatics pipeline that enables the full analyses of our quantitative micPCR/NGS data, without knowledge of command-line scripts that would normally be required. This user-friendly analytical workflow together with the validated micPCR/NGS strategy is part of the microbiota profiling platform named ‘MYcrobiota’, which has already shown promising applications in routine clinical microbiological diagnostics^15^.

In this study, the authors evaluated the utility of MYcrobiota for studying microbial dynamics within a non-chlorinated DWDS. We characterized microbial changes at consecutive locations along a full-scale DWDS in the Netherlands during a 5-month period with one-month intervals. The MYcrobiota results were compared to results of conventional microbiological analysis, including HPC, bacterial adenosine-triphosphate measurements (bATP), and intact bacterial cell concentrations assessed with FCM. Additionally, we were able to use the MYcrobiota results to detect and identify spatial and temporal bacterial dynamics within the DWDS under investigation that may be used to evaluate the success of DW treatment processes.

## Results

### Total bacterial biomass variations observed within the DWDS

**Figure 1:**
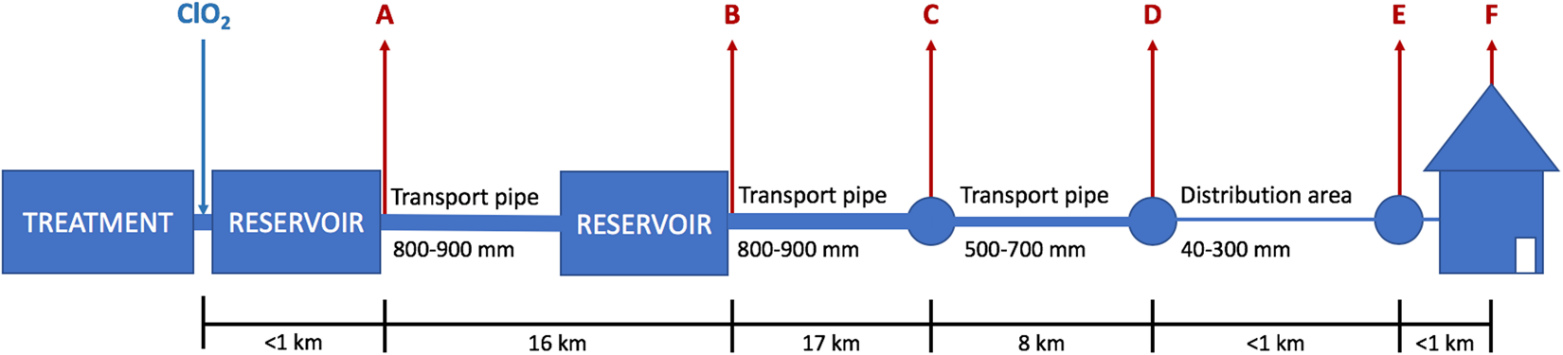
Sampling scheme. The sampling points, labelled (**A**) (effluent of water treatment plant, after chlorine dioxide dosage and after DW reservoir), (**B**) (effluent of intermediate pumping station and storage reservoir), (**C**,**D**) (transport pipelines), (**E**) (distribution pipeline), and (**F**) (tap water), are indicated by red arrows. The position of the chlorine dioxide (ClO_2_) dosage is indicated by the blue arrow. The distance between sampling points are shown in kilometres (km).

**Figure 2:**
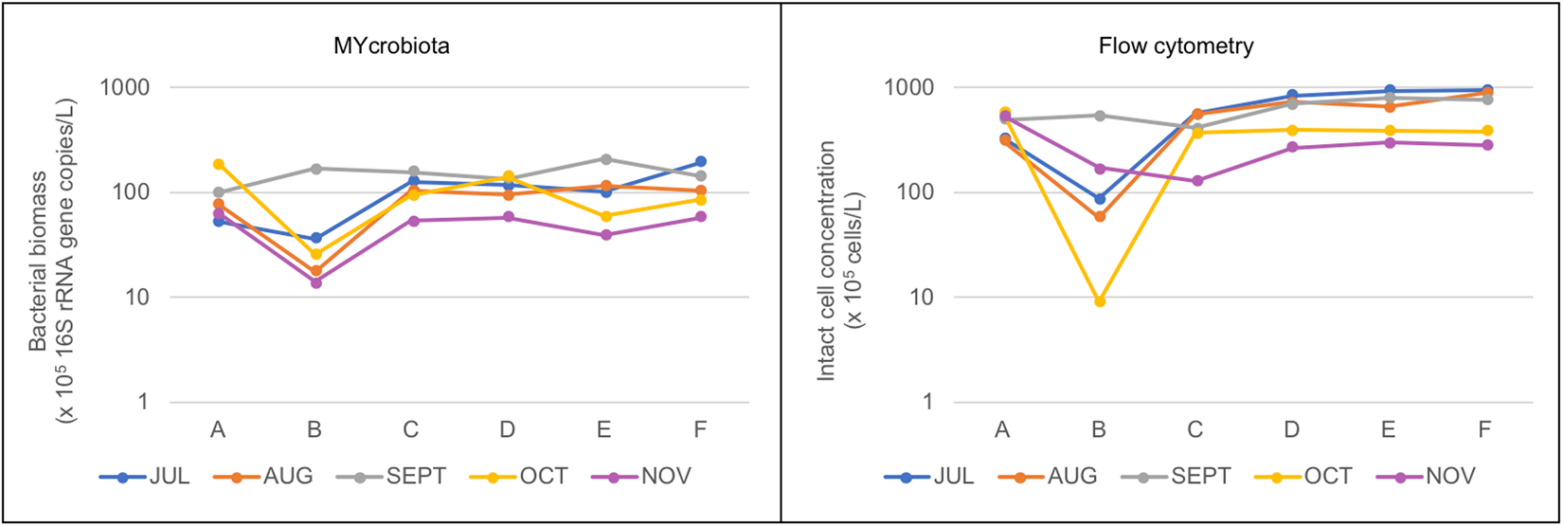
Variations of bacterial biomass measured at six consecutive locations within the DWDS. Colored lines represent 16S rRNA gene copies (left) and intact bacterial cell concentrations (right) measured from 6 consecutive locations (**A**–**F**) over a 5-month period.

A total of 30 DW samples were collected from an operational DWDS. From July till November 2016, each month the DWDS was sampled at six consecutive locations and the DW at each location processed using MYcrobiota (Fig. 1). The sequencing and quality parameters obtained are shown in Supplementary Table 1. In addition, HPC, bATP, and intact bacterial cell concentrations were measured and compared to the results obtained using MYcrobiota from all DW samples (Supplementary Table 2). These comparisons revealed that FCM and MYcrobiota were both able to detect microbiological changes within the DWDS that were not observed using HPC and bATP measurements. As shown in Fig. 2, FCM and MYcrobiota detected a clear reduction of bacterial biomass between locations A and B (with the exception of DW samples taken in September), after which time, the bacterial biomass increased towards location C, followed by a more stable bacterial biomass concentration towards location F. However, the concentration of intact bacterial cells as detected by FCM was higher in 29 out of 30 DW samples than the concentration of 16S rRNA gene copies detected with MYcrobiota, with an average of a 4.9-fold (±2.5) difference. In order to investigate the accuracy of the quantitative MYcrobiota data, we compared the number of 16S rRNA gene copies obtained using MYcrobiota with the number of 16S rRNA gene copies measured using a 16S rRNA gene quantitative PCR (qPCR)^16^. This comparison revealed an average of only a 1.3-fold (±0.3) difference between both quantitative methods, demonstrating the accuracy of MYcrobiota in determining the number of 16S rRNA gene copies in DW. In contrast, the accuracy of the FCM method could not be confirmed using complementary techniques in this study, although the standard error for FCM was previously estimated to be less than 5%^17^.

### Spatial microbial dynamics observed within the DWDS

**Figure 3:**
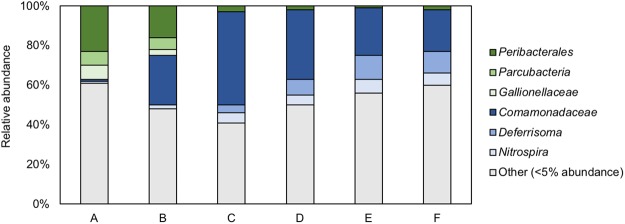
Variations in the relative abundances of six dominating OTUs detected at consecutive locations within the DWDS. The medians of 16S rRNA gene copies per OTU that were measured over a 5-month period are shown using 100% stacked bars for each consecutive location (**A**–**F**). OTUs with >5% relative abundance at the start of the DWDS are shown in shades of green, whereas OTUs with >5% relative abundance at the end of the DWDS are shown in shades of blue. All other OTUs with <5% relative abundance per sampled location were grouped together to ease visualization and are shown in grey.

The use of MYcrobiota revealed that the DWDS investigated in this study consisted of a highly diverse bacterial environment with a median of 69 (±16) OTUs – defined by 97% 16S rRNA gene sequence similarity – per DW sample (Supplementary Table 1). Only six of these OTUs were detected with a median relative abundance higher than 5% per sampled location. These dominating OTUs were classified as *Comamonadaceae*, *Deferrisoma*, *Gallionellaceae*, *Nitrospira*, *Parcubacteria*, and *Peribacterales*. Interestingly, DW samples taken at the start of the DWDS were dominated by the *Peribacterales* (median relative abundance: 23%), *Parcubacteria* (7%), and *Gallionellaceae* (7%) OTUs, whereas DW samples taken at the end of the DWDS were dominated by the *Comamonadaceae* (21%), *Deferrisoma* (11%), and *Nitrospira* (6%) OTUs as shown in Fig. 3. In contrast, 138 distinct OTUs – representing 14 different prokaryotic phyla – were detected with a median relative abundance lower than 5% per sampled location. Only 30 of these OTUs (22%) could be classified to the genus taxonomic level using public 16S rRNA gene reference databases, indicating that the vast majority of prokaryotic species present within DWDSs remain unknown or poorly understood.

**Figure 4:**
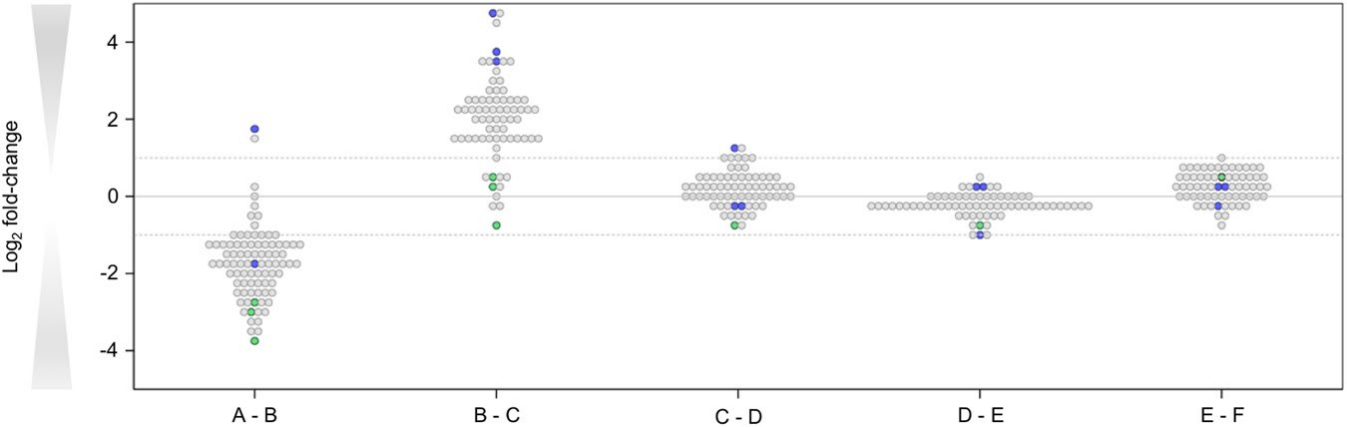
Differences in absolute abundances of OTUs detected at consecutive locations within the DWDS. OTU-level differences between DWDS locations (**A**–**F**) were calculated by dividing the number of 16S rRNA gene copies per OTU derived from two consecutive locations. For this, the medians of 16S rRNA gene copies per OTU that were measured over a 5-month period were used and their differences between two consecutive locations plotted using a binary logarithmic scale. OTUs with a relative abundance of <5% per sampled location are shown as grey dots, whereas OTUs with >5% relative abundance at location A (*Peribacterales*, *Parcubacteria*, and *Gallionellaceae*) are shown as green dots and OTUs with >5% relative abundance at location F (*Comamonadaceae*, *Deferrisoma*, and *Nitrospira*) are shown as blue dots. Differences with more than a 2-fold increase/decrease (dotted lines) were considered as significant differences that cannot be explained by technical variations introduced during sample processing.

A shift in the microbial composition tended to occur between locations A and C within the DWDS. As shown in Fig. 4, the number of 16S rRNA gene copies for the majority of OTUs decreased between locations A and B, including the dominating *Peribacterales*, *Parcubacteria*, and *Gallionellaceae* OTUs. In contrast, the number of 16S rRNA gene copies measured for most OTUs increased between locations B and C, including the dominant *Comamonadaceae*, *Deferrisoma*, and *Nitrospira* OTUs, but not the *Peribacterales*, *Parcubacteria*, and *Gallionellaceae* OTUs. Importantly, no significant increase of 16S rRNA gene copies per OTU was detected within the final stage of the DWDS (locations C to F), which includes the home plumbing system of the consumer – beyond the maintenance of the DW supply company.

### Temporal microbial dynamics observed within the DWDS

**Figure 5:**
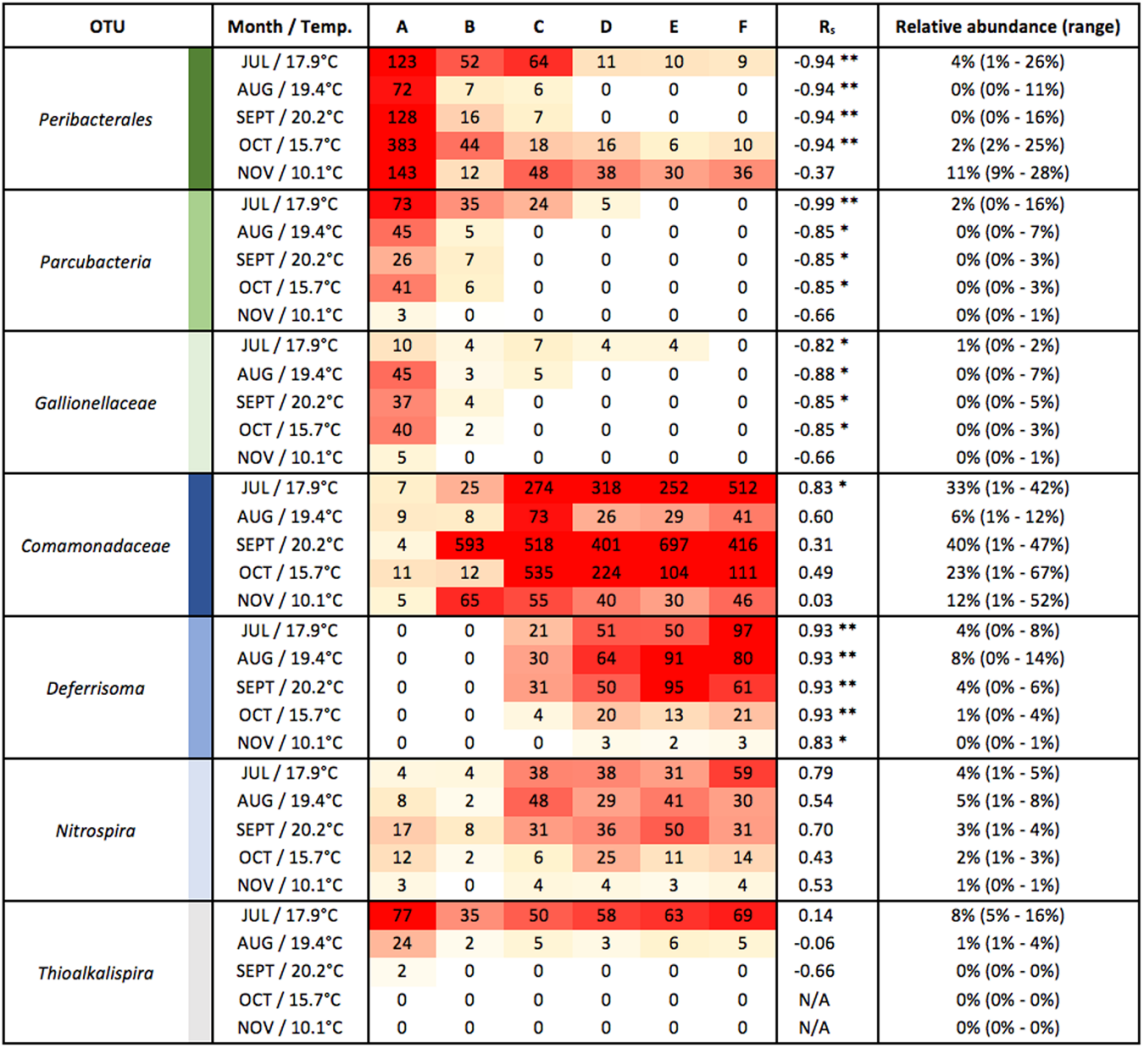
Detailed quantitative measurements of seven OTUs detected at six consecutive locations within the DWDS. The number of 16S rRNA gene copies that belong to the *Peribacterales*, *Parcubacteria*, *Gallionellaceae*, *Comamonadaceae*, *Deferrisoma*, *Nitrospira*, and *Thioalkalispira* OTUs were measured in 5 sample series and shown as a heatmap using white shades for low absolute abundances and red shades for high absolute abundances. Values within the colored boxes represent the calculated number of 16S rRNA gene copies/100 μL. Sample series are presented per month following consecutive locations within the DWDS (A-F) and includes the average water temperature that was measured at each sampled location. Spearman correlations (R_s_) and its associated significances (**p-value < 0.01; *p-value < 0.05) were calculated to check for significant increases/decreases in the absolute abundance of these OTUs throughout the DWDS. The relative abundance represents the median percentage of the number of 16S rRNA gene copies belonging to each OTU measured per sample compared to the total number of 16S rRNA gene copies measured per sample with the range given between brackets.

In order to investigate temporal trends in bacterial community structure, we compared the medians of 16S rRNA gene copies per OTU that were measured over the six consecutive DWDS locations to each month-of-sampling. From this dataset, only a single OTU – belonging to the genus of *Thioalkalispira* – could be detected during specific months only (July, August, and September, but not October and November). In contrast, the other six dominating OTUs described above were detected in all sampled months and followed the same overall dynamic trends regarding the increase and decrease in the absolute abundance of these OTUs throughout the DWDS, although these trends became weaker as water temperatures decreased (Fig. 5). Importantly, a single DW sample obtained from location B in September showed a sudden increase of *Comamonadaceae* 16S rRNA gene copies by a factor of 138 compared to location A. In fact, we measured an increase of 16S rRNA gene copies for another 42 out of 143 OTUs (30%) by at least a factor of 2 at location B in September, although their relative abundances remained below the threshold of 5%. These observations explain the deviating curve that was obtained when comparing the variations of total bacterial biomass within consecutive DW samples taken in September, namely an increase in bacterial abundance between locations A and B, while the abundance decreased at other months between these two locations (Fig. 2).

## Discussion

In this study, we evaluated the use of the user-friendly MYcrobiota platform as a culture-free monitoring tool to be used for microbiological DW quality assessment. For this, a full-scale DWDS served as a model system to investigate the performance, advantages and drawbacks of MYcrobiota compared to conventional microbiological screening methods. Our results show that MYcrobiota facilitates the accurate quantification of the total bacterial biomass present in the DWDS, as the dynamic bacterial trends – based upon the total bacterial biomass estimated using MYcrobiota – are in close agreement with the dynamic bacterial trends observed using FCM – which measures intact bacterial cell concentrations. This result indicates that MYcrobiota is not hindered by the presence of cell-free bacterial DNA derived from dead bacterial cells. Surprisingly however, the number of 16S rRNA gene copies measured using MYcrobiota was lower compared to the number of intact cells detected using FCM within the same DW samples. These differences are likely caused by a systematic loss of bacterial cells and/or bacterial DNA during sample processing (e.g. filtration and DNA extraction) since the accuracies of both methods were demonstrated in this study or elsewhere^17^. Note however that the differences in absolute quantification of intact bacterial cell concentrations and 16S rRNA gene copies did not affect the dynamic trends observed in this study.

The sensitive and accurate measurement of quantitative bacterial biomass allowed us to assess the changes in bacterial communities during water transport and distribution within a DWDS. This was illustrated by a small, but reproducible, decrease of bacterial biomass between locations A and B, which was not detected using conventional HPC and bATP measurements. The decrease of bacterial biomass is most likely the result of chlorine dioxide dosage before storage in the DW reservoir. Despite a residence time of 1 to 2 hours on average in the DW reservoir located between the chlorine dioxide dosage point and sampling location A, the disinfection process may still be happening in the subsequent transport section and intermediate reservoir located before sampling location B. This effect may be causing a decrease in bacterial biomass between locations A and B, with a decrease in intact bacterial cells, as well as a decrease in 16S rRNA gene copies of individual OTUs. Besides providing additional contact time with chlorine dioxide, the intermediate storage reservoir may also contribute to the decrease of bacterial biomass through e.g. settling, though the specific role of reservoirs was not specifically studied here. However, the disinfectant residual is not maintained in the transport and distribution system after location B, which results in an increase in bacterial biomass between locations B and C, indicating re-growth of bacteria. Importantly, further microbial re-growth could not be detected from location C to location F. The absence of changes in bacterial biomass concentration as well as community composition after location C is surprising. While locations C and D are located in large transport mains (500 to 900 mm diameter), sampling location E is situated in a small distribution pipe (152 mm diameter), and location F inside a consumer household. It has been shown earlier that small diameter pipes and low flow velocities or even stagnation have a great impact on bacterial growth and/or interaction between bulk water bacteria and biofilm^18–20^. Therefore, one would expect further growth and/or shifts in bacterial communities in these later sections. It should be noted however that DW samples were taken at location F after flushing of the taps, in order to avoid the household plumbing effect.

DW samples obtained from all locations sampled in September showed a similar number of 16S rRNA gene copies and intact bacterial cell concentrations, which suggested the presence of a stable bacterial biomass concentration across all of the locations sampled (Fig. 2). However, the use of MYcrobiota showed that these apparently stable bacterial loads actually involved unexpected increases and expected decreases of 16S rRNA gene copies derived from multiple OTUs (Fig. 5). Since the OTU dynamics between locations A and B in September were completely different compared to the general reduction of 16S rRNA gene copies measured between both locations for the other months, we speculate that this unexpected increase of bacterial biomass for certain OTUs in September was caused by deviation in the water quality leaving the treatment plant. In this period, turbidity was 2 to 3 times higher than normal in the water at location A, which suggests that the water contained more particles and/or more microorganisms. The particles and microorganisms would react with the chlorine dioxide and accelerate the chlorine dioxide decay, which is even enhanced by higher temperatures – note that the highest temperatures were measured in September throughout the whole study. A faster disinfectant decay would allow earlier re-growth in the transport pipe and/or reservoir, which would result in higher numbers of 16S rRNA gene copies and intact bacterial cell concentrations than at other times of the year. Although the reduction of bacterial biomass between locations A and B was restored in the following months, these results highlight the ability of MYcrobiota to detect specific bacterial taxa that might be linked to possible problematic scenarios, such as (sudden) excessive bacterial growth, and opens the possibility of continual monitoring and the implementation of rapid action to maintain water quality using MYcrobiota.

The MYcrobiota results indicated only a small subset of bacteria dominating the DWDS, despite its high bacterial diversity. These bacteria comprised seven distinct OTUs (at a cutoff of 97% sequence similarity) that were classified as *Comamonadaceae*, *Deferrisoma*, *Gallionellaceae*, *Nitrospira*, *Parcubacteria* (also known as candidate phylum OD1), *Peribacterales* (candidate phylum PER), and *Thioalkalispira*. All of these bacterial taxa were previously detected within other DWDS samples^2,11,21^, or within other aquatic ecosystems^22–24^. Interestingly, the *Peribacterales*, *Parcubacteria* and *Gallionellaceae* OTUs dominated the start of the DWDS, but were ‘replaced’ by the *Comamonadaceae*, *Deferrisoma*, and *Nitrospira* OTUs at the end of the DWDS. This finding was independent of the month of sampling and suggests that temporal variations are minor compared to spatial variations within this DWDS during the 5-month period of sampling. It should be noted however that most bacterial OTUs detected within this DWDS could not be identified at the genus taxonomic level due to the basic lack of 16S rRNA gene reference sequences derived from members of DW microbial communities in large publicly accessible reference databases (e.g. SILVA, RDP, GreenGenes, or NCBI). It is very likely that these OTU sequences belong to novel lineages for which there are no available culturable representatives, as the MYcrobiota protocol has been specifically established to help prevent the formation of PCR artefacts (i.e., chimeric sequences). This suggests that our current knowledge about the ecology of DW microbial communities is currently limited and requires further investigation with respect to the presence of potential (as yet unknown) bacterial pathogens in DW. Further, it is impossible to link bacterial taxa identified by MYcrobiota to their function in DWDSs. Only additional functional testing, or the identification of genes with well-established functions will provide insights into the functionality of the DW microbiome. For example, Di Rienzi and colleagues elegantly demonstrated that groundwater samples harbour non-photosynthetic bacteria belonging to a new candidate phylum sibling to *Cyanobacteria* via the use of metagenomic sequencing and metabolic reconstruction^25^.

Recently, there is a discussion about whether OTU-based strategies – such as implemented within MYcrobiota – should be replaced by newly developed amplicon sequence variants (ASV)-based methods as the standard to delineate microbial taxa^26^. These ASV-based methods avoid clustering sequences at arbitrary thresholds that define OTUs (e.g. 97%) by using only unique, identical marker gene sequences for downstream analysis. Unlike OTUs, ASVs can be resolved to single-nucleotide differences over the sequenced gene region using specialized ‘de-noising’ algorithms that is expected to increase taxonomic resolution^27^. However, Glassman and Martiny recently illustrated that OTU-based and ASV-based methods will often reveal similar ecological results when using the 16S rRNA gene^28^. This finding can be explained by the fact that 16S rRNA gene sequence types may not reflect ecologically or phylogenetically cohesive populations^29^. Therefore, in this study, we employed a traditional OTU-based strategy to simplify the identification of (sub-sets) of bacterial taxa that changed in abundance across the sampled locations. Of note, future versions of MYcrobiota could potentially include ASV-based analysis as part of its software, depending on the requirement to obtain fine-scale taxonomy results.

In conclusion, MYcrobiota is an alternative for the culture-free monitoring of bacteria that reside within DWDSs and allows DW providers to gain accurate insights into the spatial and temporal microbial dynamics within their DWDS that are not observed using conventional methods. Using information obtained by MYcrobiota facilitates the continual assessment of the desired ‘biological stability’ over the whole DWDS, thereby helping ensure that safe and high-quality DW reaches the consumer.

## Methods

### Drinking water sample collection

A total of 30 DW samples were collected from a full-scale DW transport and distribution network in the Netherlands. The transported DW was produced from surface water, using coagulation/sedimentation, rapid sand filtration, advanced oxidation, and activated carbon filtration. Chlorine dioxide (ClO_2_) was added to the water prior to storage at the DW reservoir. The residual ClO_2_ after storage is 0.03 mg/L on average, but is not maintained during DW transport and distribution. DW samples were taken at six locations throughout the DWDS, from the treatment plant towards the studied distribution area, as follows: (A) effluent of water treatment plant, after ClO_2_ dosage and after DW reservoir, (B) effluent of intermediate pumping station and storage reservoir after a first transport pipe (800–900 mm), (C and D) at sampling points in the second and third transport pipe sections (800–900 mm and 500–700 mm), (E) at a sampling point within the distribution pipe (40–300 mm) and (F) at a household tap (Fig. 1). High-density polyethylene (HD-PE) plastic bottles (Identipack BV) containing 2 mL L^−1^ of a mixed solution of sodium thiosulfate (20 g L^−1^) and of nitrilotriacetic acid (25 g L^−1^) were used to collect DW for HPC, bATP, FCM, and MYcrobiota analysis, as routinely used by accredited laboratories for DW analysis in the Netherlands. The DW samples were transported and stored at 4 °C until analysis, and processed within 24 hours after sampling.

### Conventional parameters

HPC was measured according to the Dutch standard procedure (NEN-EN-ISO 6222, 1999). In short, 1 mL per DW sample was transferred to a sterile Petri dish and mixed with 20 mL of yeast extract agar. The agar was kept at 44 °C before plating. The samples were incubated at 22 °C for 3 days. ATP was measured as described previously by Magic-Knežev and van der Kooij^30^. The ATP measurement is based on the emission of light resulting from the reaction between the ATP molecule and a luciferin/luciferase reagent (LuminATE, Celsis). For total ATP determination, ATP was first released from suspended microbial cells with nucleotide-releasing buffer (LuminEX, Celsis), while this step was not performed for the assessment of free ATP. The intensity of the emitted light was measured using a luminometer (Celsis AdvanceTM) that was calibrated with solutions of free ATP (Biotherma) in autoclaved tap water following the procedure given by the reagent manufacturer. Bacterial ATP concentrations were calculated by subtracting free ATP from total ATP concentrations. Finally, FCM analysis were performed following the protocol described by Prest *et al*.^17,31^. In short, DW samples (100 µL) were pre-heated to 37 °C for 4 minutes, stained with fluorescent dyes and incubated in the dark for 10 minutes at 35 °C before measurement. Bacterial staining with 10 µL per mL of a working solution containing a mixture of SYBR Green I (1:100 dilution in DMSO; Molecular Probes) and propidium iodide (0.5 mg/mL) was used for the assessment of intact bacterial cell concentrations. FCM measurements were performed using a BD Accuri C6 FCM (BD Accuri Cytometers) equipped with a 50 mW laser emitting at a fixed wavelength of 488 nm. The FCM is equipped with volumetric counting hardware, calibrated to measure the number of particles in a 50 µL volume fraction of a 100 µL sample. Measurements were performed at a pre-set flow rate of 35 µL per minute. A threshold value of 450 a.u. was applied on the green fluorescence channel (FL1). Bacterial signals were selected and distinguished from inorganic particles and instrument background on the BD Accuri CFlow software using electronic gating on density plots of green fluorescence (FL1; 533 nm), and red fluorescence (FL3; > 670 nm)^17^.

### MYcrobiota analysis

One liter of water from each DW sample was filtered through a 0.2 μm pore-size polycarbonate membrane filter (Sartorius) within 5 hours of sampling, using sterile (autoclaved) filtration units. The filters were stored at −20 °C until processing. DNA was extracted from the collected biomass using the PowerBiofilm DNA Isolation Kit Sample (MO BIO Laboratories) according to the manufacturer’s instructions. In addition, DNA from a sterile filter was extracted as a NEC at the same time in order to allow for the subtraction of contaminating bacterial DNA after NGS processing. Amplicon library preparation using micPCR that clonally amplified the V4 regions of 16S rRNA genes was performed as previously published^15^, but with a slight modification. In this study, the IC to determine the absolute quantity of 16S rRNA gene copies consisted of quantified genomic DNA from a *Campylobacter jejuni* bacterium (ATCC 700819). Importantly, prior to the addition of the IC, the absence of *Campylobacter* species within each DNA extract was established using a *Campylobacter* specific PCR according to Lund *et al*.^32^. We utilized the micPCR/NGS approach to process all samples, including the NEC, in triplicate in order to increase accuracy and to correct for contaminating bacterial DNA derived from the laboratory environment as previously described^14^. FASTQ-formatted sequences were extracted after paired-end sequencing of the 16S rRNA gene amplicon library using the MiniSeq system (Illumina) and processed using our previously developed bioinformatics analysis service^15^. This bioinformatics pipeline consists of 23 well-established mothur tools (v.1.36)^33^ and an additional 9 custom-made tools that have been integrated and combined in Galaxy^34^, and allows for a fully automated sequence interpretation of 16S rRNA gene micPCR/NGS data. The sequencing data that are connected to this article are uploaded to the Sequence Read Archive database with accession number SRP114562.

### Quantification of 16S rRNA gene molecules

The total number of 16S rRNA gene molecules within each DNA extract was measured using a 16S rRNA gene quantitative qPCR as described previously^16^. For this, CT-values were related to a 10-fold dilution series of a synthetic microbial community (SMC) sample, containing 10,000 16S rRNA gene copies of *Moraxella catarrhalis* (ATCC 25240), *Staphylococcus aureus* (ATCC 43300), *Haemophilus influenzae* (ATCC 10211), and *Clostridium perfringens* (ATCC 12915). The qPCRs were performed in 10 μL reaction volumes using the LightCycler 480 Probes Master (Roche) with the addition of 0.5 μM of each PCR primer and 0.25 μM of a Fam-labelled probe for the real-time detection of the 16S rRNA gene amplification. All qPCRs were performed using the following conditions: initial denaturation at 95 °C for 5 minutes followed by 45 cycles of PCR, with cycling conditions of 5 seconds at 95 °C, 10 seconds at 55 °C, and 30 seconds at 72 °C.

### Statistical analysis

Spearman’s correlation coefficients were calculated to check for significant increases/decreases in the absolute abundance of OTUs measured over the DWDS (SPSS version 23, IBM Corporation).

## Electronic supplementary material

Supplementary data
