# Supplementary material for: Monitoring of microbial dynamics in a drinking water distribution system using the culture-free, user-friendly, MYcrobiota platform
Source: Sci Rep. 2018 Oct 3;8:14727. doi: 10.1038/s41598-018-32987-x (PMC6170421; doi:10.1038/s41598-018-32987-x)
Supplement: Supplementary file 1 — Supplementary data [file 41598_2018_32987_MOESM1_ESM.pdf]

Monitoring of microbial dynamics in a drinking water distribution system using the culture-free, user-friendly, MYcrobiota platform.

Stefan A. Boers, Emmanuelle I. Prest, Maja Taučer-Kapteijn, Aleksandra Knezev, Peter G. Schaap, John P. Hays, Ruud Jansen

Supplementary Table 1. Sequence characteristics of all thirty drinking water samples processed in triplicate during this study.

Description	MYcrobiota results*
# raw sequence reads (range)	131,742 (14,326 – 213,990)
# raw sequence reads used for analysis	10,000
- % removed sequence reads that does not match 100% with primers	13.7 (± 0.5)
- % removed sequence reads that are shorter than 200 and longer than 300 bases	0.8 (± 0.5)
- % removed sequence reads containing ambiguous bases (N)	2.5 (± 0.2)
- % removed sequence reads that did not align to the SILVA reference alignment release 123	0.2 (± 0.1)
- % removed sequence reads that were identified as chimeric sequences	3.0 (± 1.5)
- % removed sequence reads that were classified as non-prokaryotic OTUs	0.1 (± 0.2)
# sequence reads clustered into OTUs at 97% similarity (range)	7,938 (7,257 – 8,307)
# observed OTUs at 97% similarity	69 (± 16)
% sequence reads clustered into the *Campylobacter* (IC) OTU**	7.7 (± 1.8)

*: All data represent median values obtained from 30 drinking water samples that were processed in triplicate using MYcrobiota. The standard deviation is given in parentheses, unless stated otherwise. **: Prior to micPCR amplification, *Campylobacter* DNA was added as internal calibrator (IC) in such a concentration that each drinking water sample contained approximately 10% of the IC 16S rRNA gene copies. The IC is used to calculate a correction factor that in turn is used to convert the obtained sequence reads per OTU to 16S rRNA gene copies per OTU.

Supplementary Table 2. Comparison of microbial dynamics in time and distance over the studied DWDS based on conventional methods and MYcrobiota.

Month / location	Temp. (°C)	HPC (CFU/mL)	bATP (ng/L)	FCM (intact bacterial cells/mL)	MYcrobiota (16S rRNA gene copies/mL)
July					
Location A	18.5	<25	< 2.0	33,400	5,292
Location B	18.7	<25	< 2.0	8,700	3,630
Location C	17.9	328	2.6	57,000	12,670
Location D	17.0	92	4.8	83,200	11,877
Location E	17.4	33	4.8	92,300	10,227
Location F	17.8	<25	3.9	94,200	19,282
August					
Location A	19.7	<25	< 2.0	31,200	7,678
Location B	20.0	<25	< 2.0	5,800	1,764
Location C	19.4	37	< 2.0	55,900	10,357
Location D	18.7	39	3.7	73,800	9,466
Location E	18.8	92	< 2.0	66,600	11,513
Location F	19.5	46	< 2.0	89,400	10,470
September					
Location A	20.9	<25	< 2.0	49,500	9,954
Location B	20.9	<25	2.5	54,200	17,092
Location C	20.2	<25	3.5	41,000	15,715
Location D	19.4	28	3.3	70,100	13,393
Location E	19.6	<25	3.0	80,400	21,141
Location F	20.1	29	4.3	76,800	14,387
October					
Location A	15.0	<25	< 2.0	58,800	18,946
Location B	15.2	<25	< 2.0	900	2,566
Location C	15.1	<25	3.3	37,100	9,393
Location D	16.0	<25	4.1	39,600	14,110
Location E	16.1	<25	< 2.0	39,200	6,019
Location F	16.6	<25	2.0	38,000	8,526
November					
Location A	8.7	<25	< 2.0	54,200	6,383
Location B	10.0	<25	< 2.0	17,200	1,400
Location C	9.9	25	2.2	12,900	5,445
Location D	10.6	<25	< 2.0	26,300	5,784
Location E	10.7	<25	< 2.0	29,800	3,919
Location F	10.9	<25	< 2.0	28,000	5,787

Drinking water samples were taken at six consecutive locations within the DWDS (A - pump station, B - storage reservoir, C and D - transport pipelines, E - distribution pipeline, F - tap water) over a 5-month period. HPC – heterotrophic plate counts, bATP – bacterial adenosine-triphosphate measurement, FCM – flow cytometry.
